# Physical activity and non-suicidal self-injurious behavior in Chinese adolescents: the chain mediating role of psychological capital and relative deprivation

**DOI:** 10.3389/fpsyt.2024.1509967

**Published:** 2024-11-29

**Authors:** Yingzhe Gao, Changfen Lu, Xiaoqiang Zhang, Beining Han, Huijuan Hu

**Affiliations:** ^1^ School of Physical Education, Central China Normal University, Wuhan, China; ^2^ School of Physical Education, Wuhan Sports University, Wuhan, China; ^3^ School of Physical Education, Anhui Normal University, Wuhu, China

**Keywords:** physical activity, non-suicidal self-injury, psychological capital, relative deprivation, adolescents

## Abstract

**Introduction:**

Physical activity has been shown to alleviate negative emotions. We examined whether physical activity is associated with lower non-suicidal self-injurious behavior in adolescents and the mediating and chain-mediating roles of psychological capital and relative deprivation in this association.

**Methods:**

451 secondary school students (44.57% girls; ages 13-19) completed the Physical Activity Rating Scale, Adolescent Non-Suicidal Self-Injurious Behavior Scale, Psychological Capital Scale, and Adolescent Relative Deprivation Scale in their classrooms. In addition, this study used SPSS 26.0 for statistical data analysis and the SPSS macro program PROCESS 4.1 to explore the mediation role.

**Results:**

Regression-based analyses showed that higher psychological capital and lower relative deprivation individually and sequentially mediated the association between physical activity and lower NSSI.

**Conclusion:**

These findings contribute to a deeper understanding of how and why physical activity affects adolescents’ non-suicidal self-injurious behaviors. At the same time, the result may provide new insights into prevention and intervention efforts for non-suicidal self-injurious behaviors in adolescents.

## 1 Introduction

Non-suicidal self-injury (NSSI) is the direct, deliberate, and repetitive injury of one’s body tissues without suicidal intent and social or cultural acceptance (1). Adolescents are facing increasing pressure from society, school, and family, and have become vulnerable to NSSI behaviors (2). According to the survey, adolescents have a high prevalence of NSSI, about 17% to 18% of adolescents have NSSI behaviors globally (3), and the incidence of NSSI among Chinese adolescents is 24.7% (4). Negative emotions and aggressive feelings are considered risk factors for NSSI (5). NSSI, in turn, is behaviors are a significant predictor of suicidal behaviors (6) and a risk factor for lower social stability (7), school safety (8), family harmony (9), and healthy growth (10). Specifically, NSSI increases adolescents’ risk of anxiety, depression, substance abuse, affective disorders, and many other negative psychological and behavioral outcomes (11, 12). Self-injurious behavior therefore remains a complex and dangerous public mental health problem despite its non- or low lethality (13). Self-injurious behavior, especially in adolescents, requires urgent attention from researchers and clinicians.

Physical activity is part of an active, healthy lifestyle that promotes physical and mental health (14). Several factors have been shown to contribute to these effects, including increases in dopamine, serotonin, and brain-derived neurotrophic factor (BDNF) in the brain (15, 16), and a sense of accomplishment (17). Physical activity has also been shown to be a protective factor against psychological distress (18). It has been shown to have an inhibitory and channeling effect on negative thoughts and emotions (19). It has also been shown to be associated with lower NSSI (20, 21). However, little research (22, 23)has been conducted on the intrinsic mechanisms of the relationship between physical exercise and lower NSSI, and further systematic examination is needed to advance research progress on adolescent NSSI behaviors. In the current study, we tested two psychological variables, namely psychological capital and relative deprivation, as potential chain mediators of the association between physical activity and lower non-suicidal self-injury. The results could provide a theoretical and practical basis for developing prevention and intervention programs to reduce NSSI among Chinese adolescents.

### 1.1 The relationship between physical activity and non-suicidal self-injurious behavior

Physical activity has been shown to be negatively associated with NSSI behaviors in several studies (20). In one study, adolescents were assigned to one of two physical activity groups. The first group engaged in low physical activity (no physical activity over 60 minutes at a time in the last week) and high sedentary behavior (more than two hours of sedentary time per day last week); the second group engaged in high physical activity (physical activity of more than 60 minutes on three or more occasions in the last week) and low sedentary behavior (sedentary time less than one hour per day last week). Compared to the first group, the second group showed fewer symptoms of depression, anxiety, and incidents of NSSI at the end of the study (23). Another study further analyzed the relationship between the frequency of physical activity and NSSI behavior. The study found a dose-response relationship between physical activity and NSSI behaviors in adolescents, with girls who never exercised being approximately 2 to 2.5 times more likely to engage in NSSI behaviors compared to girls who exercised daily (24). Finally, a cross-sectional study of 1,675 depressed adolescents between the ages of 12 and 18 found that the total effect of physical activity on NSSI was positive and significant, but that excessive physical activity (≥ 480 minutes per week) led to an increase in NSSI in depressed adolescents (25). Based on the results of these studies, we proposed the following hypothesis.

Hypothesis 1. Physical activity will be significantly and negatively associated with non-suicidal self-injurious behavior in adolescents.

### 1.2 Psychological mediators of the link between physical activity and lower NSSI

Cognitive-behavioral theories emphasize the interplay between an individual’s thoughts, emotions, and behaviors (26). In the current study, we tested the role of thoughts in the association between physical activity and NSSI. Some have argued that physical activity can help the individual identify and change negative thought patterns in a process called cognitive restructuring (27). We assume that physical activity promotes cognitive restructuring, which in turn reduces the occurrence of NSSI behaviors triggered by emotional distress (28). In the current study, we tested two aspects of cognitive restructuring as potential chain mediators of the association between physical activity and lower NSSI, namely greater psychological capital and lower relative deprivation.

#### 1.2.1 The mediating role of psychological capital

Psychological capital constitutes the psychological resources of optimism, hope, self-efficacy, and resiliency (29). Optimism is a person’s confidence that things will turn out to be positive for them; hope is their thought that they can achieve what they want; self-efficacy is the belief that they could succeed when faced with challenging tasks; and resiliency is their ability to persevere and work hard to succeed in the face of difficulty and adversity. Physical activity has been shown to contribute to adolescents’ psychological capital (30, 31). The cross-sectional study found that physical activity not only significantly and positively predicted psychological capital (32), but that psychological capital also mediated the relationship between physical activity and variables such as social anxiety (33) and well-being (34), and that psychological capital also moderated the effect of moderating physical activity on mobile phone dependence (34). A 16-week intervention study found that self-efficacy, resiliency, optimism dimension scores and total psychological capital scores were higher in the intervention group than in the control group when they engaged in moderate-intensity physical activity (35). Some of the constituent dimensions of psychological capital and associated positive psychological factors are thought to slow the development of NSSI behavior. One study of a group of 659 high school students documented that all four of the components of psychological capital (optimism, hope, self-efficacy, and resilience) were significantly and negatively correlated with NSSI behavior (36). In another study, hope as a component of psychological capital, attenuates the association between depressive symptoms and non-suicidal self-injury in female adolescents and plays a protective role between feelings of discrimination and non-suicidal self-injury in middle school students. A multicohort simultaneous comparative modeling study of college students found higher rates of NSSI in the low psychological capital group compared to the high psychological capital group (37). Indirect evidence of the effect of psychological capital comes from a study on the psychological characteristic of psychological resilience, defined as mental toughness, and negatively correlated with NSSI frequency (38). Accordingly, we proposed the following hypothesis.

Hypothesis 2. Psychological capital will mediate the relationship between physical activity and non-suicidal self-injury in adolescents.

#### 1.2.2 The mediating role of relative deprivation

Social comparison theory suggests that people have an intrinsic motivation to evaluate their opinions, abilities, and attitudes, and they tend to do so by comparing themselves to others (39). The term relative deprivation has been used to describe a subjective cognitive and emotional experience in which an individual or group experiences negative emotions, such as anger and resentment when they perceive themselves to be at a disadvantage in comparison with a reference group (40). Physical activity contributes to the development of an individual’s capacity for self-affirmation (41) and has a role in reducing an individual’s sense of relative deprivation (42). People perceive an equal right to participate in physical activity by comparing themselves with others, which helps to enhance their social support network (43) and thus inhibits the generation of relative deprivation. As athletic ability improves, the person can gain a sense of achievement and self-worth in comparison with others, thus reducing the individual’s sense of relative deprivation (44).

The negative emotions associated with the perception of relative deprivation are thought to catalyze the development of extreme behavior (45), such as anger, jealousy and disappointment can trigger adolescents to participate in social crimes or attack others (46). Only one study has tested the association between relative deprivation and NSSI. High school students’ perception of relative deprivation was directly correlated with NSSI, and also mediated the association between upward social network comparison and NSSI (47). Other studies have not focused on NSSI, but on the co-morbid problem of suicidal ideation (48). A study of Chinese university students confirmed that the higher the individual’s perception of relative deprivation, the higher the suicidal ideation (49). Another study found that the perception of relative deprivation had not only a significant direct effect on suicidal ideation but also an indirect effect through the perception of lower social support and negative core self-evaluation (50). It is inferred that adolescents’ participation in physical activity reduces the perception of relative deprivation and thus lowers the risk of NSSI behaviors. We proposed the following hypothesis.

Hypothesis 3. Relative deprivation will mediate the relationship between physical activity and non-suicidal self-injury in adolescents.

#### 1.2.3 The chain mediation roles of psychological capital and relative deprivation

Research on the relationship between psychological capital and relative deprivation in adolescents is still in its infancy, but there is empirical of this relationship. Several studies have shown that different elements of psychological capital are associated with lower relative deprivation. For example, studies on mental resilience training documented an ameliorative effect on relative deprivation (51), and the positive effects of training on mental toughness (40), hope (52), and gratitude (53) were significantly negatively correlated with relative deprivation. While these studies focused on specific elements of psychological capital, it may be more valuable to study the integration of different elements. According to the theory of multiple resources (54), the elements of psychological capital work in a synergistic manner, and the synergistic effect has a greater impact than the effect of a single factor. Consistent with this viewpoint, we treated psychological capital as a composite variable in the current study. Thus, the indirect pathway through which physical activity influences non-suicidal self-injury in adolescents may be physical activity → higher psychological capital → lower sense of relative deprivation → lower non-suicidal self-injury. Accordingly, we proposed the following hypothesis.

Hypothesis 4. Psychological capital and relative deprivation will sequentially mediate the association between physical activity and non-suicidal suicidal behavior in adolescents.

**Figure 1:**
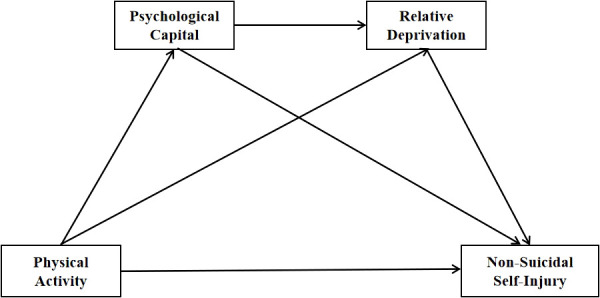
The proposed conceptual model.

Together, the hypotheses constitute tests of a conceptual model in which adolescents’ physical activity is associated with lower non-suicidal self-injury through the sequential mediating effects of higher psychological capital and lower relative deprivation. The model is presented in **Figure 1**.

## 2 Materials and methods

### 2.1 Research design

This is an empirical study analyzing the results using quantitative methods. Adolescents completed self-report questionnaires of physical activity, psychological capital, relative deprivation, and non-suicidal self-injury. These cross-sectional data were used to test statistical models of mediation.

### 2.2 Participants

The adolescents in this study were enrolled in a secondary school (three years of junior high school and three years of high school) in Dalian City, China. Informed consent was obtained from the parents, and all students participated voluntarily. All students knew that their data would be used in the study and that they had the option to stop responding to the questionnaires at any time. The questionnaires were administered in the students’ classrooms. A trained proctor gave the instructions. At the end of the administration, the questionnaires were collected by the main examiner.

In August 2024, the questionnaire was distributed to 500 secondary school students who completed questionnaires in their classrooms. After deleting questionnaires with irregular responses and excessive missing values, there were 451 valid questionnaires, with a validity rate of 90.20%. Of the 451 secondary school students, there were 250 (55.43%) boys and 201 (44.57%) girls; their ages ranged from 13 to 19 years old (M ± SD = 14.69 ± 1.41 years); and there were 214 (47.45%) in their second year of junior high school and 237 (52.5%) in the third year of junior high school. The study was approved by the Research Ethics Committee of the first author’s institution.

### 2.3 Measurement

#### 2.3.1 Physical activity

The Physical Activity Rating Scale (PARS-3) was used to measure physical activity during the past month, then that Liang (1994) developed a Chinese-language version (55). The scale assesses the amount of activity on three dimensions: frequency, intensity, and duration. Items include “How many times a month do you do physical activity”, “How intense is your physical activity”, and “How many minutes at a time do you do the above physical activities”. Ratings are made using a Likert 5-point scale, the frequency scored 1 (less than 10 minutes at a time) to 5 (60 minutes or more per session), with higher scores indicating more frequent activity, the intensity scored 1 (light activity) to 5 (high intensity and sustained activity), with higher scores indicate more intense activities and the duration scored 0 (less than once a month) to 4 (once or twice a week). According to the formula of physical activity behavior = intensity × time × frequency, the highest score was 100 points and the lowest score was 0 points. Evaluation criteria: ≤ 19 points for low activity; 20-42 points for moderate activity; ≥ 43 points for high activity, with higher scores indicating greater physical activity. The Cronbach’s α coefficient for the scale in this study was.745.

#### 2.3.2 Non-suicidal self-injury

NSSI within one year was measured using the self-report Adolescent Non-Suicidal Self-Injurious Behavior Scale (Wan et al., 2018) (56), a 12-item measure developed for use in the Chinese cultural context, sample items include “scratch yourself on purpose”, and “bite yourself on purpose”. Each item is rated on a 5-point Likert scale from 0 (none) to 4 (always). Total scores range from 0-48, with higher scores indicating more frequent non-suicidal self-injurious behavior in adolescents. The Cronbach’s α coefficient for the scale in this study was.961.

#### 2.3.3 Psychological capital

The Positive Psychological Capital Inventory (Luthans et al., 2007) is a self-report measure of psychological capital. We used the Chinese version of this measure (Zhang et al., 2010) (57), which consists of 26 items measuring four dimensions of psychological capital: optimism, hope, self-efficacy, and resilience, sample items include “I always see the good side of things”, “I pursue my goals with confidence”, “I enjoy taking on difficult and challenging work” and “When I get frustrated, I recover quickly”. A 7-point Likert scale was used to rate each item from 1 (not at all) to 7 (completely). Five of the questions were reverse scored (questions 8, 10, 12, 14, and 25 resulted in reverse scores), and the average score for all items was calculated, with higher scores indicating higher individual psychological capital. The Cronbach’s α coefficient for this scale in this study was.950.

#### 2.3.4 Relative deprivation

The Adolescent Relative Deprivation Scale (Tian, 2021) (58) was used to assess the level of relative deprivation in adolescents. The Chinese-language self-report scale consists of 10 items representing two dimensions, namely cognitive relative deprivation and emotional relative deprivation. The adolescents were asked to rate how the perception described in each item was in line with their thinking. Each item is rated on a 5-point Likert scale from 1 (very much not in line with) to 5 (very much in line with), and the higher the total score, the higher the degree of relative deprivation. The Cronbach’s alpha coefficient of this scale in this study was.922.

### 2.4 Statistical analysis

The study applied SPSS 26.0 to test generate descriptive statistics and correlations, common method bias, and test questionnaire reliability. The bias-corrected percentile bootstrap (Sampling times 5000) method was then used to test mediation, using the PROCESS 4.1 macro developed by Hayes (59) for use in SPSS 26.0. All variables were standardized before model testing.

## 3 Results and analyses

### 3.1 Common method variance

Because all variables were assessed through self-report, Harman’s single-factor test was used to assess possible common method bias (60). Exploratory factor analysis yielded eight factors with eigenvalues greater than 1, the first of which explained 33.68% of the variance. This value is lower than the critical value of 40%, so it can be judged that the results were not seriously affected by common method bias.

### 3.2 Descriptive statistics and correlation analysis

As shown in **Table 1**, physical activity was positively correlated with psychological capital (*r* = .15, *p* <.01), and negatively correlated with relative deprivation (*r* = -.28, *p* <.01) and non-suicidal self-injury (*r* = -.22, *p* <.01). In addition, psychological capital was negatively correlated with relative deprivation (*r* = -.54, *p* <.01) and non-suicidal self-injury (*r* = -.41, *p* <.01). Relative deprivation was positively correlated with non-suicidal self-injury (*r* = .35, *p* <.01). These correlations were in line with the proposed conceptual model.

**Table 1:** Descriptive statistics and correlations among study variables (N = 451).

Variable	M	SD	1	2	3	4
1. PA	28.09	29.09				
2. PC	128.56	28.50	.15**			
3. RD	22.94	8.80	-.28**	-.54**		
4. NSSI	4.01	8.12	-.22**	-.41**	.35**	

**p <.01; M, mean; SD, standard deviation; PA, physical activity; PC, psychological capital; RD, relative deprivation; NSSI, non-suicidal self-injury.

### 3.3 Tests of hypotheses

First, we tested the study variables for multicollinearity. VIF values were all less than 10, which can be judged as demonstrating no multicollinearity problem. Second, previous studies have shown that age and gender are important factors influencing adolescents’ non-suicidal self-injurious behaviors (61), in terms of age, a systematic review of longitudinal studies has demonstrated that the prevalence of NSSI peaks around mid-adolescence (around 15-16 years of age) and declines in late adolescence (around 18 years of age) (62); and in terms of gender, the majority of the studies have pointed to a higher prevalence of NSSI among girls than boys in the adolescent population (63, 64); therefore, we transformed age and gender into dummy variables and entered them as control variables in all analyses.

As shown in **Table 2**, regression analyses showed that physical activity significantly positively predicted psychological capital (β = .16, *p* <.001); when physical activity and psychological capital were tested as simultaneous predictors of relative deprivation, both had a significant negative predictive effect (β = -.22, *p* <.001; β = -0.50, *p* <.001); when physical activity, psychological capital, and relative deprivation were tested simultaneously as predictors of non-suicidal self-injury, physical activity and psychological capital were significant negative predictors (β = -.13, *p* <.01; β = -.32, *p* <.001), and relative deprivation was a significant positive predictor (β = .14, *p* <.01).

**Table 2:** Results of regression analyses (N = 451).

Regression Equation		Overall Fit Coefficient			Significance of Regression Coefficient		95% Confidence Interval	
Outcome	Predictor	*R*	*R* ^2^	*F*	β	*t*	LLCL	ULCL
NSSI	Age	.22	.05	7.97	-.01	-.21	-.03	.02
	Gender				-.03	-.66	-.25	.13
	PA				-.23	-4.73^***^	-.33	-.13
PC	Age	.16	.27	4.08	.01	.37	-.22	.03
	Gender				.14	1.41	-.05	.39
	PA				.16	3.33^***^	.07	.26
PRD	Age	.58	.33	55.61	-.01	-.79	-.03	.01
	Gender				-.15	-1.91	-.31	.01
	PA				-.22	-5.31^***^	-.30	-.14
	PC				-.50	-12.73^***^	-.58	-.42
NSSI	Age	.46	.21	23.86	.01	.03	-.02	-.03
	Gender				.01	.14	-.16	.19
	PA				-.13	-2.91^**^	-.23	-.04
	PC				-.32	-6.36^***^	-.42	-.22
	RD				.14	2.78^**^	.04	.25

**p <.01, ***p <.001. PA, physical activity; PC, psychological capital; RD, relative deprivation; NSSI, non-suicidal self-injury.

**Figure 2:**
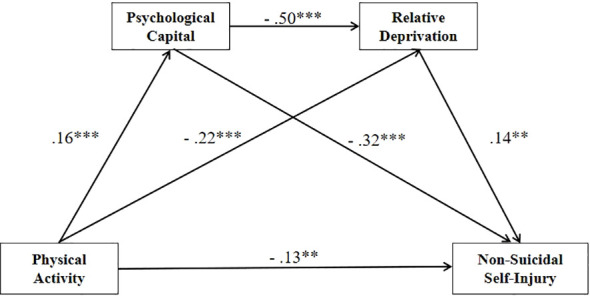
The chain mediation model. **p < .01; ***p < .001.

Further, a chain mediation model was tested using physical activity as the independent variable, non-suicidal self-injury as the dependent variable, and psychological capital and relative deprivation as the chain mediating variables (**Figure 2**). It was found that physical activity, psychological capital, and relative deprivation all had significant direct effects on non-suicidal self-injury behavior.

In addition, the mediating roles of adolescents’ psychological capital and relative deprivation in the association between physical activity and non-suicidal self-injury were tested using Model 6 of the SPSS macro program PROCESS(2013). Effect sizes were estimated using 5,000 bootstrap samples. Age and gender were controlled in all analyses. A mediation effect is statistically significant when the 95% confidence interval does not include 0. The results are shown in **Table 3**.

**Table 3:** Mediation effects of psychological capital and lower relative deprivation in the association between physical activity and lower NSSI.

Model	Path	Effect	Boot SE	Boot LLCI	Boot ULCI	Proportion of variance
Direct Effect	PA→NSSI	-.13	0.05	-.23	-.04	58.67%
	PA→PC→NSSI	-.05	.02	-.10	-.02	22.56%
Indirect Effects	PA→RD→NSSI	-.03	.01	-.06	-.01	13.68%
	PA→PC→RD→NSSI	-.01	.01	-.03	-.01	5.10%
Total Effect		-.23	.05	-.33	-.13	100%

SE, standard error; PA, physical activity; PC, psychological capital; RD, relative deprivation; NSSI, non-suicidal self-injury. Boot SE = standard error of the mean boot estimate; LLCI = lower limit of the 95% confidence interval (95% CI); ULCI = upper limit of the 95% CI. The mediation effect is significant when the confidence interval does not contain 0.

The results showed that the direct effect of physical activity on NSSI was significant (standardized effect value = -.13, accounting for 58.67% of the total effect). Hypothesis 1 was supported. Psychological capital had a significant indirect effect on this association (standardized effect value = -.05, accounting for 22.56% of the total effect). Hypothesis 2 was supported. Relative deprivation was also a significant mediator (standardized effect value = -.03, accounting for 13.68% of the total effect). Hypothesis 3 was supported. The indirect effect explained by the chain mediators of psychological capital and relative deprivation was significant (standardized effect value = -.01, accounting for 5.10% of the total effect). Hypothesis 4 was supported. Together, the indirect effects explained 41.33% of the total effect.

## 4 Discussion

### 4.1 Relationship between physical activity and non-suicidal self-injury

Physical activity has been shown to promote mental health. In the current study, we explored the negative relationship between physical activity and non-suicidal self-injury (NSSI) using cognitive-behavioral theory as a conceptual framework. We tested whether the association between physical exercise and lower NSSI could be explained based on the mediating effects of higher psychological capital (e.g., hope and optimism) and lower relative deprivation (e.g., lower belief that one is at a disadvantage). There was a significant negative correlation between physical activity and non-suicidal self-injury—that is, the higher the level of physical activity among adolescents, the lower the risk of non-suicidal self-injury. The results of regression analysis further indicated that physical activity had a significant negative predictive effect on non-suicidal behavioral self-injury, a finding that supports the cognitive-behavioral theory and is consistent with previous studies (22–25).

It is the individual’s perception of the event that leads to emotional and behavioral responses (65). Adolescents might perceive NSSI as a way to relieve stress, cope with trauma, and seek acceptance (66), but these perceptions need to be changed because of the danger presented by NSSI behaviors. Physical activity can promote positive cognitions through positive body-environment interactions, which in turn can effectively circumvent NSSI (28). Specifically, physical activity can help adolescents to develop a more positive self-image and self-evaluation (67), so that they can see their progress and achievements and see themselves and the world around them more positively, thus reducing the occurrence of NSSI behaviors (68) Yu et al. ‘s study point out that perceived social support is the mediating variable of physical activity reducing NSSI behavior in adolescents. This result provides support for our interpretation of the direct predictive role of physical activity on adolescent NSSI behavior. Therefore, we infer that physical activity is often accompanied by group social activities, and these social interactions can enhance adolescents’ social support networks, contributing to a more positive cognitive model and may reduce the probability and frequency of non-suicidal self-injurious behaviors (69). This suggests that schools and parents should cultivate adolescents’ interest in physical activity and help them develop physically and mentally by reconstructing positive cognitions and strengthening social support networks.

### 4.2 Psychological capital mediates the relationship between physical activity and non-suicidal self-injury

Our findings suggest that the positive psychological effects of physical activity may enrich adolescents’ psychological capital, in turn inhibiting the occurrence of adolescents’ NSSI. First, the finding that physical activity significantly and positively predicts adolescent psychological capital is consistent with previous research (32–34, 70). The positive promoting effect of physical exercise on psychological capital may come from many aspects. On a psychological level, this positive feedback enhances an individual’s self-efficacy when they are successful in sporting activities and competitions (71). This appears to be the primary mechanism by which physical activity improves psychological capital. However, there are also some positive effects of physical activity that contribute less to psychological capital, especially in terms of physical functioning improvements (72). This is because the enhancement of the cardiovascular, musculoskeletal, and respiratory systems by physical activity is more at the level of physical fitness than directly at the level of psychological capital formation.

Second, the research by Giordano et al. (73). showed that coping skills are a form of NSSI prevention. we assert that the link between psychological capital and lower NSSI is due to better coping. Although coping is not part of psychological capital, it may be related to several elements of psychological capital. People with higher psychological capital tend to have more effective coping strategies to effectively manage negative emotions and stress (74). More specifically, individuals with a high sense of self-efficacy are more likely to adopt positive coping styles to solve problems and seek social support because they believe they can cope with challenges (75); individuals with a strong sense of hope tend to have a positive attitude towards the future and believe they can find solutions to problems (76); optimistic people tend to see the positive side of things and maintain a positive coping attitude even in the face of adversity (77); and individuals with high resiliency can maintain a stable mindset in the face of stress and challenges and recover quickly from adversity (78). In summary, positive coping can lead to better coping with stress and frustration through cognitive remodeling, which may help to reduce adolescent NSSI behaviors due to negative thought patterns.

### 4.3 Relative deprivation mediates the relationship between physical activity and non-suicidal self-injury

The study also confirmed the mediating role of relative deprivation in the association between physical activity and adolescent non-suicidal self-injurious behaviors. First, physical activity had a significant negative effect on adolescents’ relative deprivation. This is the only study to date that has explored the relationship between physical activity and relative deprivation in adolescents. According to the Social Comparison Theory (39), adolescents’ participation in physical activity, especially team activities, enhances an individual’s sense of social identity and allows them to focus more on their contributions within the group rather than on comparisons with the outside, thus reducing their sense of relative deprivation. This result is somewhat consistent with Taub’s research, which suggests that physical activity is effective in increasing an individual’s sense of social identity (79). Thus, the intrinsic mechanism by which physical activity reduces adolescents’ sense of relative deprivation may be due to a heightened sense of social identity.

Second, NSSI behaviors and suicidal ideation are highly comorbid, so relative deprivation may be a risk factor for adolescent NSSI behavior because of its link to suicide ideation (80, 81). The Experiential Avoidance Model suggests that negative emotions are an important factor in triggering an individual’s NSSI behavior (82). Feelings of relative deprivation may cause individuals to experience intense emotional distress, such as anger, depression, etc (53). NSSI behaviors may be used as a means of emotion regulation to alleviate these negative emotions. This suggests that schools, communities, teachers, and parents should emphasize and encourage adolescents to be physically active to enhance their social identity and divert attention from feelings of relative deprivation. These may be beneficial in reducing adolescents’ NSSI behaviors and suicidal ideation.

### 4.4 Chain mediation roles of psychological capital and relative deprivation

The results suggest that physical activity can influence adolescent non-suicidal self-injurious behavior by increasing psychological capital, which in turn could reduce the perception of relative deprivation. Among the results, the significant negative predictive effect of psychological capital on relative deprivation is consistent with Tao et al. who argued that they believe psychological capital moderates the effects of relative deprivation on psychologically problematic behaviors (83). Psychological capital can help adolescents cope with difficulties and setbacks in life more proactively (84), recover more quickly from failures (85), continue to pursue their goals and adopt positive coping strategies to deal with life’s stresses and challenges (86). These strengths mean that individuals tend to have positive expectations about the future and may help adolescents focus less on the gap between themselves and others, thereby reducing the sense of relative deprivation resulting from failing to meet social standards or expectations. By contrast, low psychological capital may lead to greater feelings of relative deprivation. From previous research, it is clear that individuals with low psychological capital may have difficulty coping with life’s challenges, recovering from setbacks and having a negative view of the future (87). This leads them to feel deprived of similar opportunities or achievements when they see others successfully overcoming difficulties (53).

Furthermore, it is important to note that the independent mediating effect values for psychological capital and relative deprivation were higher compared to the chain-mediation values. This suggests that while focusing on the chain-mediation effects we should also pay attention to the independent mediating role of the two between physical activity and NSSI. In conclusion, the chain-mediation effects of psychological capital and relative deprivation provide a deeper explanation of the mechanisms by which physical activity influences non-suicidal self-injury, expanding research in the fields of clinical psychology and behavioral health.

## 5 Implications

This paper validates a chain-mediated model of physical activity reducing non-suicidal self-injurious behavior in adolescents by increasing psychological capital and diminishing relative deprivation. On the one hand, based on cognitive-behavioral theory, this study found that physical activity can both directly reduce adolescent non-suicidal self-injurious behaviors by promoting positive cognitive development, and indirectly reduce adolescent non-suicidal self-injurious behaviors by improving the individual’s psychological capital so that the individual has a positive coping style. On the other hand, based on the social comparison theory and the NSSI experiential avoidance model, it was found that physical activity weakened the relative deprivation of adolescents in social comparison by strengthening the social support network and individual psychological capital, and indirectly reduced adolescents’ non-suicidal self-injurious behaviors by decreasing the negative emotion of relative deprivation, which is a validation of the theoretical model mentioned above.

The results of this study have implications for the development of prevention programs for non-suicidal self-injurious behaviors among adolescents worldwide, especially in China. On the one hand, this study found that physical activity significantly and negatively predicted non-suicidal self-injurious behavior among adolescents. This suggests that the Chinese government should invest more in school and community sports facilities, that the Chinese education authorities should require schools to diversify sports activities to stimulate adolescents’ interest in sports, and that Chinese parents should be encouraged to support their children’s participation in sports. On the other hand, it was found that psychological capital and relative deprivation had a chain mediating effect between physical activity and non-suicidal self-injury. This suggests that Chinese teachers and parents should enhance positive motivation for adolescents, avoid excessive comparisons, and create an equal and harmonious classroom and family environment to reduce adolescents’ non-suicidal self-injurious behaviors by increasing psychological capital and decreasing relative deprivation. In addition, this study can inform the development of non-suicidal self-injury prevention programs for adolescents in countries with more severe self-injurious behaviors and lower levels of psychiatric care.

## 6 Limitations and future directions

This study has some limitations that need to be further refined in future studies. First, the questionnaire used in this study was a self-assessment scale, and the data collected came from adolescents’ self-reports, which may have issues with subjectivity and reliability. Future research could collect data from multiple subjects such as teachers, parents, and peers. Second, although the chain mediation model constructed in this study helped to understand the relationship between physical activity, psychological capital, relative deprivation, and non-suicidal self-injury, the study was inherently cross-sectional. Longitudinal studies or clinical intervention studies could be used in the future to further explore the causal relationships between variables. Finally, this study did not assess the effect of psychotherapy and counseling interventions, a potential protective factor, on adolescents’ non-suicidal self-injurious behavior. Future studies should further focus on the subjects’ psychotherapy and counselling interventions, which would help to exclude external factors affecting the results of the study and strengthen the scientific validity of the study.

## 7 Conclusions

The results showed that physical activity was significantly negatively associated with non-suicidal self-injury among adolescents. In addition, physical activity appeared to reduce adolescent non-suicidal self-injurious behaviors by enhancing psychological capital and by weakening relative deprivation. Further, the chain mediation effect was significant: physical activity was associated with higher psychological capital, which in turn predicted lower relative deprivation, which predicted lower NSSI. Thus, these mediators acted independently and sequentially in the association between physical activity and NSSI.

## Data Availability

The raw data supporting the conclusions of this article will be made available by the authors, without undue reservation.
